# Mediterranean Diet and Physical Activity Decrease the Initiation of Cardiovascular Drug Use in High Cardiovascular Risk Individuals: A Cohort Study

**DOI:** 10.3390/antiox10030397

**Published:** 2021-03-05

**Authors:** Margarita Ribó-Coll, Sara Castro-Barquero, Camille Lassale, Emilio Sacanella, Emilio Ros, Estefanía Toledo, José V. Sorlí, Andrés Díaz-López, José Lapetra, Carlos Muñoz-Bravo, Fernando Arós, Miquel Fiol, Lluis Serra-Majem, Xavier Pinto, Olga Castañer, César I. Fernández-Lázaro, Olga Portolés, Nancy Babio, Ramón Estruch, Álvaro Hernáez

**Affiliations:** 1 August Pi i Sunyer Biomedical Research Institute (IDIBAPS), 08036 Barcelona, Spain; mribocoll@gmail.com (M.R.-C.); sacastro@clinic.cat (S.C.-B.); esacane@clinic.cat (E.S.); eros@clinic.cat (E.R.); restruch@clinic.cat (R.E.); 2 PhD Program in Food Science and Nutrition, Faculty of Pharmacy and Food Science, Universitat de Barcelona, 08028 Barcelona, Spain; 3 Department of Medicine, Faculty of Medicine and Health Sciences, University of Barcelona, 08036 Barcelona, Spain; 4 Consorcio CIBER, M.P. Fisiopatología de la Obesidad y Nutrición (CIBEROBN), Instituto de Salud Carlos III, 28029 Madrid, Spain; classale@imim.es (C.L.); etoledo@unav.es (E.T.); jose.sorli@uv.es (J.V.S.); andres.diaz@urv.cat (A.D.-L.); joselapetra543@gmail.com (J.L.); aborau@secardiologia.es (F.A.); miguel.fiol@ssib.es (M.F.); lserra@dcc.ulpgc.es (L.S.-M.); xpinto@bellvitgehospital.cat (X.P.); ocastaner@imim.es (O.C.); cflazaro@unav.es (C.I.F.-L.); olga.portoles@uv.es (O.P.); nancy.babio@urv.cat (N.B.); 5 Cardiovascular Risk and Nutrition Research Group, Hospital del Mar Medical Research Institute (IMIM), 08003 Barcelona, Spain; 6 Internal Medicine Service, Hospital Clínic, 08036 Barcelona, Spain; 7 Lipid Clinic, Endocrinology and Nutrition Service, Hospital Clínic, 08036 Barcelona, Spain; 8 Department of Preventive Medicine and Public Health, Universidad de Navarra, 31008 Pamplona, Spain; 9 Navarra Institute for Health Research (IdiSNA), 31008 Pamplona, Spain; 10 Department of Preventive Medicine and Public Health, Universidad de Valencia, 46010 Valencia, Spain; 11 Unitat de Nutrició i Salut Pública, Departament de Ciències Mèdiques Bàsiques, Universitat Rovira i Virgili, 43201 Reus, Spain; 12 Institut d’Investigació Sanitaria Pere Virgili (IISPV), 43204 Reus, Spain; 13 Department of Family Medicine-Research Unit, Distrito Sanitario Atención Primaria Sevilla, 41013 Sevilla, Spain; 14 Department of Public Health and Psychiatry, Universidad de Málaga, 29071 Málaga, Spain; carlosmb@uma.es; 15 Department of Cardiology, Hospital Universitario de Álava, 01009 Vitoria, Spain; 16 Health Research Institute of the Balearic Islands (IdISBa), Hospital Son Espases, 07120 Palma de Mallorca, Spain; 17 Instituto de Investigaciones Biomédicas y Sanitarias, Universidad de Las Palmas de Gran Canaria, 35016 Las Palmas, Spain; 18 Centro Hospitalario Universitario Insular Materno Infantil, Servicio Canario de Salud, 35016 Las Palmas, Spain; 19 Lipids and Vascular Risk Unit, Internal Medicine, Hospital Universitario de Bellvitge, 08907 L’Hospitalet de Llobregat, Spain; 20 Unitat de Nutrició Humana, Departament de Bioquimica i Biotecnologia, Universitat Rovira i Virgili, 43201 Reus, Spain; 21 Blanquerna School of Health Sciences, Universitat Ramon Llull, 08025 Barcelona, Spain; 22 Centre for Fertility and Health, Norwegian Institute of Public Health, 0473 Oslo, Norway

**Keywords:** mediterranean diet, physical activity, glucose-lowering drugs, antihypertensive drugs, statins, fibrates, antiplatelet drugs, vitamin K epoxide reductase inhibitors, antianginal drugs, cardiac glycosides

## Abstract

Our aim was to assess whether long-term adherence to a Mediterranean diet (MedDiet) and leisure-time physical activity (LTPA) were associated with a lower initiation of cardiovascular drug use. We studied the association between cumulative average of MedDiet adherence and LTPA and the risk of cardiovascular drug initiation in older adults at high cardiovascular risk (PREvención con DIeta MEDiterránea trial participants) non-medicated at baseline: glucose-lowering drugs (*n* = 4437), antihypertensives (*n* = 2145), statins (*n* = 3977), fibrates (*n* = 6391), antiplatelets (*n* = 5760), vitamin K antagonists (*n* = 6877), antianginal drugs (*n* = 6837), and cardiac glycosides (*n* = 6954). One-point increases in MedDiet adherence were linearly associated with a decreased initiation of glucose-lowering (HR: 0.76 [0.71–0.80]), antihypertensive (HR: 0.79 [0.75–0.82]), statin (HR: 0.82 [0.78–0.85]), fibrate (HR: 0.78 [0.68–0.89]), antiplatelet (HR: 0.79 [0.75–0.83]), vitamin K antagonist (HR: 0.83 [0.74; 0.93]), antianginal (HR: 0.84 [0.74–0.96]), and cardiac glycoside therapy (HR: 0.69 [0.56–0.84]). LTPA was non-linearly related to a delayed initiation of glucose-lowering, antihypertensive, statin, fibrate, antiplatelet, antianginal, and cardiac glycoside therapy (minimum risk: 180–360 metabolic equivalents of task-min/day). Both combined were synergistically associated with a decreased onset of glucose-lowering drugs (*p*-interaction = 0.04), antihypertensive drugs (*p*-interaction < 0.001), vitamin K antagonists (*p*-interaction = 0.04), and cardiac glycosides (*p*-interaction = 0.01). Summarizing, sustained adherence to a MedDiet and LTPA were associated with lower risk of initiating cardiovascular-related medications.

## 1. Introduction

Better adherence to a Mediterranean Diet (MedDiet) and regular practice of leisure-time physical activity (LTPA) are both able to prevent major cardiovascular clinical outcomes [1,2]. Beneficial effects of MedDiet and LTPA may be mediated by improvements in risk factors related to blood pressure, glucose and lipid metabolism, oxidative stress, and low-grade inflammation [3,4]. However, there is limited information on the effect of lifestyle factors on the need of or delay in cardiovascular drug use. Regarding dietary modifications, only two studies are available and focused on antidiabetic therapy. A MedDiet intervention was associated with a decreased risk of initiating glucose-lowering therapy in non-treated type 2 diabetes patients in the PREDIMED (PREvención con DIeta MEDiterránea) study [5], and another intervention with a low-carbohydrate MedDiet-like dietary pattern was related to a reduced need of these medications in middle-aged adults [6]. In relation to physical activity, beyond the association between LTPA and a greater discontinuation of antidiabetic treatment [7] or a reduced risk of warfarin bleeding complications [8], only one cohort study in a Scandinavian general population has reported that higher LTPA levels are linked to a decreased risk of initiating antihypertensive medication [9].

High adherence to a MedDiet together with high LTPA levels has been associated with a decreased risk of cardiovascular events [10] and all-cause mortality [11]. Short-term, small-scale intervention studies recommending a combination of a MedDiet-like dietary pattern and LTPA have been related to decreases in body weight, blood pressure, fasting glucose, insulin resistance, total cholesterol, and triglyceride levels [12]. In addition, interim analyses of the first year of a large-scale intervention with a combination of hypocaloric MedDiet with LTPA and behavioral support in the PREDIMED-Plus study have confirmed these benefits and reported a decrease in the levels of leptin and low-grade inflammation biomarkers [13]. However, the combined effect of MedDiet and LTPA on the necessity of cardiovascular drugs, and a potential synergistic protection, is still unknown. Therefore, the aim of this study was to assess the prospective association of high adherence to a MedDiet and LTPA levels, alone and combined, with the risk of initiating cardiovascular drug use in Spanish older adults at high cardiovascular risk.

## 2. Materials and Methods

### 2.1. Study Population

This work is a prospective analysis using information about participants of the PREDIMED trial as a cohort study. The PREDIMED trial was a randomized controlled trial conducted in Spain between 2003 and 2009 to assess the effects of a nutritional intervention fostering the adherence to a MedDiet on the primary prevention of cardiovascular outcomes in an older population at high cardiovascular risk. Eligible volunteers were men (aged 55–80 years) and women (aged 60–80 years) without cardiovascular disease at enrolment but with type 2 diabetes or at least three of the following cardiovascular risk factors: hypertension (systolic blood pressure ≥ 140 mmHg, diastolic blood pressure ≥ 90 mmHg, or using antihypertensive drugs), high concentrations of low-density lipoprotein cholesterol (≥160 mg/dL), low levels of high-density lipoprotein cholesterol (<40 mg/dL in men, <50 mg/dL in women), body mass index > 25 kg/m^2^, smoking, and family history of premature coronary disease (<55 years old in first degree male relatives, <65 years old in first degree female relatives). The study protocol complied with the Declaration of Helsinki, was approved by Institutional Review Boards of all recruiting sites, was registered under the International Standard Randomized Controlled Trial Number ISRCTN35739639 (http://www.isrctn.com/ISRCTN35739639, accessed on 28 January 2021), has been previously described in detail elsewhere [1,14], and is available in the PREDIMED study website (http://www.predimed.es, accessed on 28 January 2021). All participants provided written informed consent before joining the trial.

**Figure 1:**
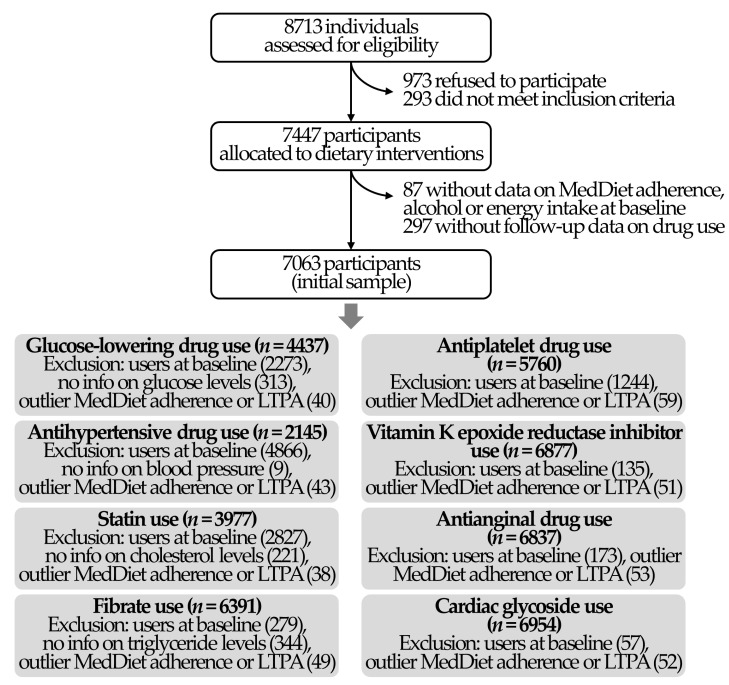
Study flowchart. LTPA: leisure-time physical activity; MedDiet: Mediterranean diet.

For these analyses, we used the PREDIMED data as an observational prospective cohort, adjusting all analyses for intervention groups. Of the 7447 randomized participants, we excluded 87 with no available baseline data on MedDiet adherence, alcohol intake, or energy consumption, and 297 without information on cardiovascular drug use at follow-up visits, yielding an analytical sample of 7063 individuals. Of note, analyses of initiation of cardiovascular-related drugs were performed in non-users at study entry, so we also excluded users of each specific drug at baseline. Furthermore, we excluded volunteers with no information on baseline levels of fasting glucose (*n* = 313), systolic blood pressure (*n* = 9), total cholesterol (*n* = 221), and triglycerides (*n* = 344) in the analyses related to the initiation of glucose-lowering drugs, antihypertensive medication, statins, and fibrates, respectively. The study flowchart is available in Figure 1.

### 2.2. Outcomes

At baseline and yearly follow-up visits, we collected data on the use (yes/no) of the main cardiovascular-related drugs. Glucose-lowering drugs included biguanides, thiazolidinediones, sulfonylureas, meglitinides, glucagon-like peptide analogues/agonists, dipeptidyl peptidase-4 inhibitors, α-glycosidase inhibitors, and insulin. Antihypertensive therapies included renin-angiotensin-aldosterone system inhibitors, calcium antagonists, thiazide/thiazide-like/loop diuretics, mineralocorticoid receptor antagonists, beta-blockers, alpha-blockers, and other minor antihypertensive families. Lipid-lowering drug use was defined as any use of statins or fibrates. Antiplatelet drugs included acetylsalicylic acid as antiplatelet, cilostazol, clopidogrel, dipyridamole, ditazol, ticlopidine, and triflusal. When assessing the use of other anticoagulants, we registered any use of vitamin K epoxide reductase inhibitors (warfarin and acenocumarol) (the use of other anticoagulants, such as direct oral anticoagulant drugs, was marginal in the follow-up period of our study). Antianginal drugs included nitrates (nitroglycerin, isosorbide mononitrate, molsidomine) and other angina pectoris drugs (trimetazidine, ivabradine, ranolazine). Finally, we registered any use of cardiac glycosides.

Using these data, we defined incidence of the initiation of any of these therapies among baseline non-users. “Initiation” was defined as the occurrence of the initiation of medication use that lasted until the last visit of the volunteer [5]. Regarding doubtful cases, we only considered as valid outcomes any initiation of medication that persisted for at least three subsequent follow-up visits and was not based on more than one visit in which the use of the drug was not reported.

### 2.3. Exposure Variables

We estimated MedDiet adherence at each visit using the MedDiet adherence score. It was a short screener questioning whether the volunteer followed 14 essential dietary traits related to a MedDiet, validated in Spanish adults. The consumption of the following scored positively: (1) olive oil as main fat for cooking/seasoning; (2) ≥4 tablespoons/day of olive oil; (3) ≥2 servings/day of vegetables; (4) ≥3 servings/day of fruit; (5) ≥3 servings of mixed nuts (30 g) per week; (6) ≥3 servings of legumes (150 g, boiled) per week; (7) ≥3 servings/week of fish or seafood; (8) wine in moderation (100 mL/day on average, within meals); (9) <1 serving/day of red and processed meat; (10) poultry and rabbit over red and processed meat; (11) <1 serving/day of butter, margarine, or cream; (12) <1 carbonated or sugar-sweetened beverage/day; (13) <2 servings/week of non-homemade pastries or sweets; and (14) a “sofrito”-based dish at least twice per week [15].

LTPA was estimated by the self-administered Minnesota Leisure-Time Physical Activity Questionnaire, previously validated in Spanish men and women [16,17]. The questionnaire reported the number of days and min/day that participants performed 67 different activities in the previous year. LTPA was quantified in metabolic equivalents of task-minute per day (METs-min/d) by multiplying the metabolic equivalents of task linked to an activity with its mean duration in min/day.

### 2.4. Covariates

Trained personnel collected baseline data on: age; sex; educational level; prevalence of diabetes, hypercholesterolemia, hypertriglyceridemia, and hypertension; systolic blood pressure; body mass index; and smoking habit [1,14]. Glucose, total cholesterol, and triglyceride levels were assessed in local laboratories in overnight fasting plasma samples collected at baseline and stored at −80 °C until analysis. From a validated 137-item food frequency questionnaire, we estimated the consumption of alcohol (in g/day) and energy (in kcal/day) [14].

### 2.5. Power Analyses

The number of total individuals and cases that occurred during follow-up allowed to detect as significant (*α =* 0.05), with a power ≥80%, hazard ratios (HR) for the comparisons between low MedDiet adherence + low LTPA levels and high MedDiet adherence + high LTPA levels ranging from 0.38 (cardiac glycoside) to 0.80 (statin) (Table S1). This calculation was performed using the “powerSurvEpi” package in R Software [18].

### 2.6. Statistical Analyses

Baseline characteristics were expressed as means and standard deviations (normally distributed continuous variables), medians and interquartile ranges (non-normally distributed continuous variables), and proportions (categorical variables).

We used Cox proportional hazards regression models with restricted cubic splines to evaluate graphically the non-linear associations of the cumulative mean of MedDiet adherence and of LTPA levels with the risk of starting to use cardiovascular-related medications [19]. We calculated the cumulative mean of MedDiet adherence or LTPA as the average of all adherence score/LTPA values until the occurrence of the outcome (incident cases) or the last study visit with available data (non-cases). Any participant with outlier values in cumulative means of MedDiet adherence score (<5 points) or LTPA levels (>1000 METs-min/day) was excluded. We set the reference cut-point at the minimum value for each exposure variable (5 points of MedDiet adherence score, 0 METs-min/day of LTPA). We defined follow-up time as the time between the date of enrolment and: (1) the midpoint between the last visit in which the volunteer did not use the medication and the first visit in which the volunteer reported its use; or (2) 1 December 2010, whichever came first. Models were stratified by sex, recruitment site, and educational level (primary/secondary/higher/unavailable), and adjusted for baseline: age (continuous), diabetes (yes/no), hypercholesterolemia (yes/no), hypertriglyceridemia (yes/no), hypertension (yes/no), smoking habit (current/former/never), body mass index (continuous), alcohol consumption (continuous), energy intake (continuous), and PREDIMED intervention group. MedDiet adherence analyses were further adjusted for LTPA (continuous), and LTPA analyses for MedDiet adherence score (continuous). We substituted the following covariates in the models: diabetes for fasting glucose (continuous) in glucose-lowering therapy initiation analyses, hypertension for systolic blood pressure in antihypertensive therapy initiation analyses, hypercholesterolemia for total cholesterol (continuous) in statin initiation analyses, and hypertriglyceridemia for triglycerides (continuous) in fibrate initiation analyses. We used robust variance estimators to account for intra-cluster correlations in all survival analyses [1].

To study the combined effect of diet and physical activity, we first classified volunteers according to their cumulative means of MedDiet adherence and LTPA values (“low”—below the media—or “high”—above the median—values). We then classified the participants in four categories: low values in both exposures (reference), low MedDiet adherence and high LTPA, high MedDiet adherence and low LTPA, and high MedDiet adherence and LTPA. We tested whether high MedDiet adherence and high LTPA levels were synergistically associated with lower risk of drug initiation by applying a likelihood ratio test between the Cox models with and without the interaction product-term “cumulative MedDiet adherence x cumulative LTPA.”

We performed the analyses using the “survival” package in R Software (version 3.5.2) [20,21].

## 3. Results

### 3.1. Study Population

Study volunteers were elderly adults (67 years old on average, 58% women) with high prevalence of cardiovascular risk factors at baseline (83% hypertension, 72% hypercholesterolemia, 49% diabetes, 47% obesity, 14% current smokers) (Table 1). The number of persons at risk of new drug use and median follow-up times were as follows for each drug-specific analysis: glucose-lowering drugs (*n* = 4437, 4.0 years), antihypertensive drugs (*n* = 2145, 2.6 years), statins (*n* = 3977, 3.7 years), fibrates (*n* = 6391, 4.8 years), antiplatelet drugs (*n* = 5760, 4.0 years), vitamin K epoxide reductase inhibitors (*n* = 6877, 4.5 years), antianginal drugs (*n* = 6837, 4.6 years), and cardiac glycosides (*n* = 6954, 4.8 years). Among the individuals susceptible to initiate medication, the use of antidiabetics was initiated in 16.9% of the participants, antihypertensive drugs in 46.2%, statins in 30.0%, fibrates in 2.16%, antiplatelet drugs in 19.1%, vitamin K epoxide reductase inhibitors in 3.00%, antianginal drugs in 2.47%, and cardiac glycosides in 0.82%.

### 3.2. MedDiet Adherence and Risk of Cardiovascular Drug Initiation

**Figure 2:**
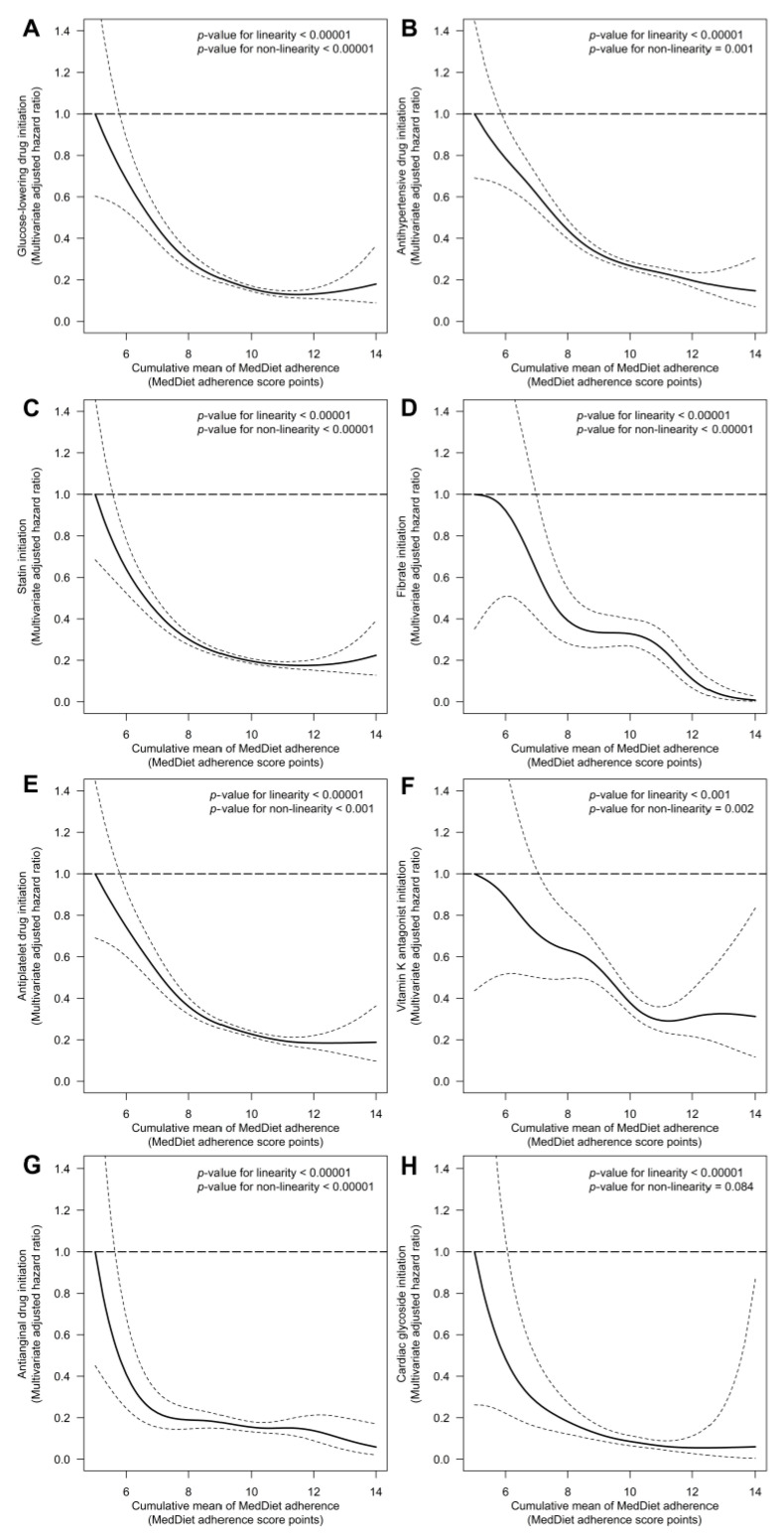
Association between long-term adherence to a MedDiet and the risk of initiating glucose-lowering (**A**), antihypertensive (**B**), statin (**C**), fibrate (**D**), antiplatelet (**E**), vitamin K epoxide reductase inhibitors (**F**), antianginal (**G**), and cardiac glycoside (**H**) therapies. Cox proportional hazards regression models with cubic splines were stratified by sex, recruitment site, and educational level, and adjusted for: age, diabetes, hypercholesterolemia, hypertriglyceridemia, hypertension, smoking habit, body mass index, alcohol consumption, energy intake, leisure-time physical activity, and PREDIMED intervention group. We substituted diabetes for fasting glucose in the analysis on glucose-lowering therapy initiation, hypertension for systolic blood pressure in the analysis on antihypertensive therapy initiation, hypercholesterolemia for total cholesterol in the analysis on statin initiation, and hypertriglyceridemia for triglycerides in the analysis on fibrate initiation. We used robust variance estimators to account for intra-cluster correlations.

One-point increases in cumulative MedDiet adherence were linearly associated with the following decreases in the risk of drug initiation: 24% for glucose-lowering therapy (HR: 0.76 [95% CI: 0.71; 0.80]), 21% for antihypertensive medication, (HR: 0.79 [0.75; 0.82]), 18% for statins (HR: 0.82 [0.78; 0.85]), 22% for fibrates (HR: 0.78 [0.68; 0.89]), 21% for antiplatelet drugs (HR: 0.79 [0.75; 0.83]), 17% for vitamin K epoxide reductase inhibitors (HR: 0.83 [0.74; 0.93]), 16% for antianginal drugs (HR: 0.84 [0.74; 0.96]), and 31% for cardiac glycosides (HR: 0.69 [0.56; 0.84]) (Figure 2).

### 3.3. LTPA Levels and Risk of Cardiovascular Drug Initiation

**Figure 3:**
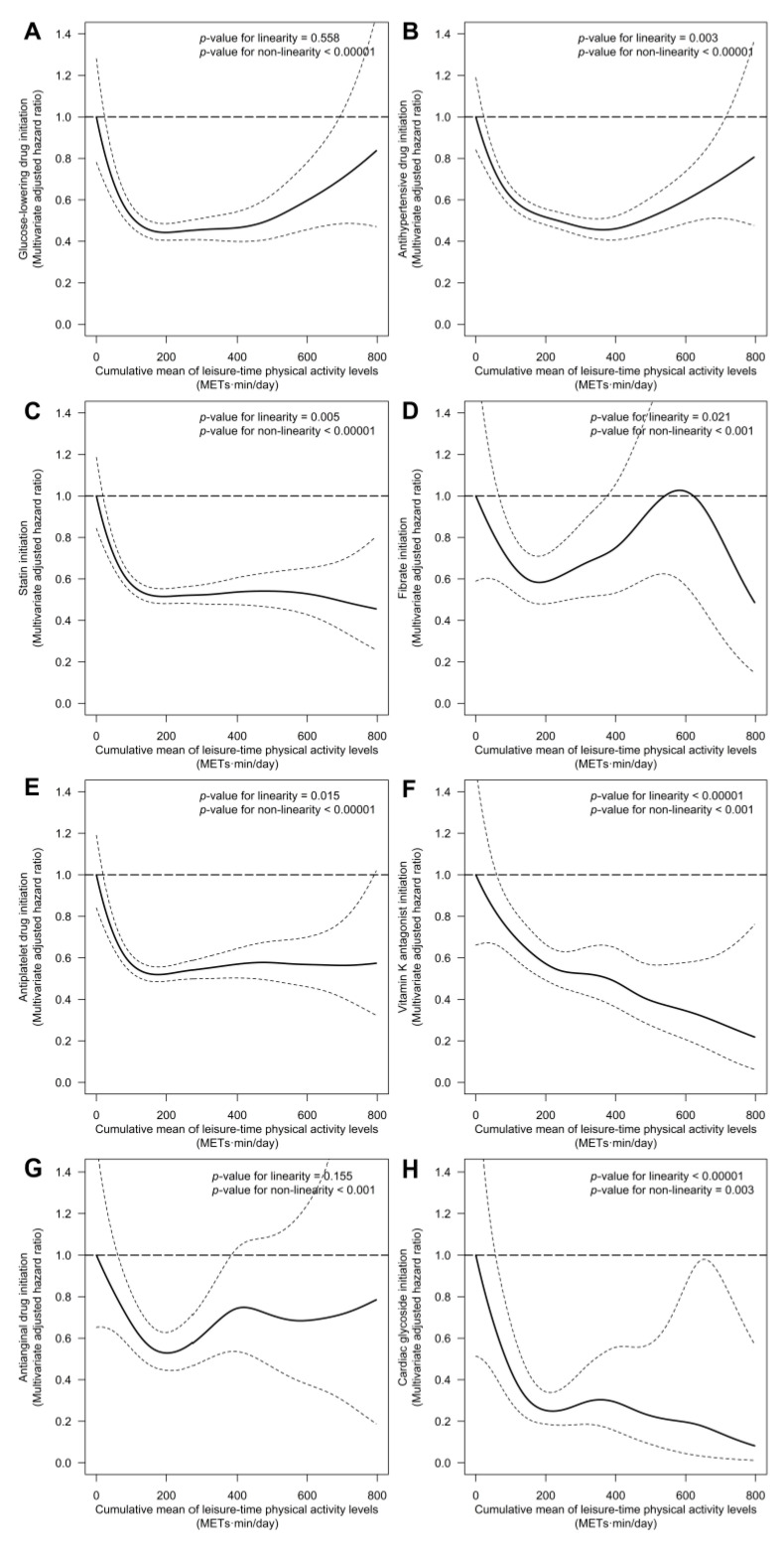
Association between long-term levels of physical activity and the risk of initiating glucose-lowering (**A**), antihypertensive (**B**), statin (**C**), fibrate (**D**), antiplatelet (**E**), vitamin K epoxide reductase inhibitors (**F**), antianginal (**G**), and cardiac glycoside (**H**) therapies. Cox proportional hazards regression models with cubic splines were stratified by sex, recruitment site, and educational level, and adjusted for: age, diabetes, hypertension, hypercholesterolemia, hypertriglyceridemia, smoking habit, body mass index, alcohol consumption, energy intake, adherence to a MedDiet, and PREDIMED intervention group. We substituted diabetes for fasting glucose in the analysis on glucose-lowering therapy initiation, hypertension for systolic blood pressure in the analysis on antihypertensive therapy initiation, hypercholesterolemia for total cholesterol in the analysis on statin initiation, and hypertriglyceridemia for triglycerides in the analysis on fibrate initiation. We used robust variance estimators to account for intra-cluster correlations.

LTPA levels were only linearly linked to lower initiation risk of vitamin K epoxide reductase inhibitors (100 METs-min/day increments were related to 10% less risk, HR: 0.90 [0.81; 1.00]). We observed non-linear associations of LTPA with the risk of initiation of the rest of cardiovascular drugs (Figure 3). The lowest risk values were observed between 180 and 360 METs-min/day (relative to 0 METs-min/day) for the following drug families: glucose-lowering drugs (lowest risk at 198 METs-min/day, HR: 0.44 [0.40; 0.48]), antihypertensive drugs (at 364 METs-min/day, HR: 0.47 [0.42; 0.52]), statins (at 204 METs-min/day, HR: 0.51 [0.48; 0.55]), fibrates (at 188 METs-min/day, HR: 0.63 [0.52; 0.77]), antiplatelet drugs (at 176 METs-min/day, HR: 0.52 [0.49; 0.56]), antianginal drugs (at 202 METs-min/day, HR: 0.53 [0.45; 0.63]), and cardiac glycosides (at 220 METs-min/day (HR: 0.25 [0.18; 0.34]).

### 3.4. Adherence to a MedDiet Combined with LTPA Levels and Risk of Cardiovascular Drug Initiation

**Figure 4:**
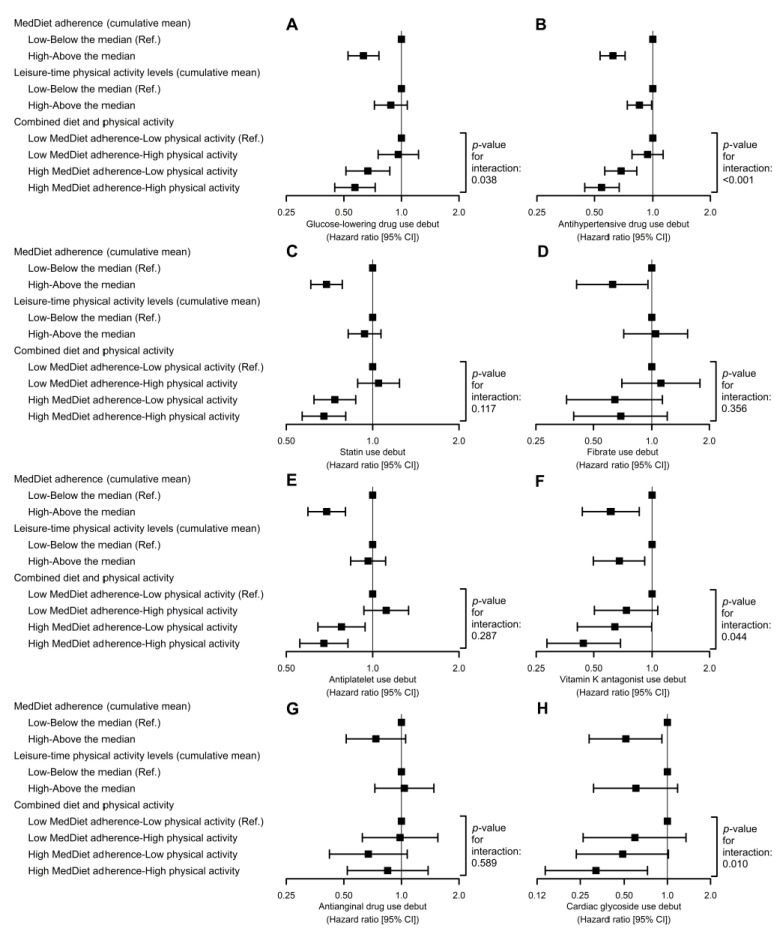
Combination of MedDiet adherence and leisure-time physical activity and the risk of initiating glucose-lowering (**A**), antihypertensive (**B**), statin (**C**), fibrate (**D**), antiplatelet (**E**), vitamin K epoxide reductase inhibitors (**F**), antianginal (**G**), and cardiac glycoside (**H**) therapies. Cox proportional hazards regression models were stratified by sex, recruitment site, and educational level, and adjusted for: age, diabetes, hypercholesterolemia, hypertriglyceridemia, hypertension, smoking habit, body mass index, alcohol consumption, energy intake, and PREDIMED intervention group. We substituted diabetes for fasting glucose in the analyses on glucose-lowering therapy initiation, hypertension for systolic blood pressure in the analyses on antihypertensive therapy initiation, hypercholesterolemia for total cholesterol in the analyses on statin initiation, and hypertriglyceridemia for triglycerides in the analyses on fibrate initiation. We used robust variance estimators to account for intra-cluster correlations.

High MedDiet adherence and LTPA levels had a synergistic impact on the risk of initiating the following therapies: glucose-lowering drugs (*p*-interaction = 0.04), antihypertensive drugs (*p*-interaction < 0.001), vitamin K epoxide reductase inhibitors (*p*-interaction = 0.04), and cardiac glycosides (*p*-interaction = 0.01) (Figure 4).

## 4. Discussion

In older individuals at high cardiovascular risk, we observed that adherence to a MedDiet and LTPA of at least approximately 200 METs-min/day were associated with a decreased necessity of cardiovascular-related medications. In addition, the combination of MedDiet and LTPA was synergistically associated with a delayed initiation of glucose-lowering, antihypertensive, vitamin K epoxide reductase inhibitor, and cardiac glycoside therapies.

Several cardiometabolic benefits of a traditional MedDiet have been described in observational and clinical studies [3]. Our findings extend this protective association to cardiovascular-related medications and suggest a delayed initiation of glucose-lowering, antihypertensive, statin, fibrate, antiplatelet, vitamin K epoxide reductase inhibitor, antianginal, and cardiac glycoside therapies in individuals with high adherence to a MedDiet. Our results agree with previously reported associations of MedDiet with a decreased risk of initiating glucose-lowering therapy in type 2 diabetes patients [5] and vitamin K epoxide reductase inhibitors in high cardiovascular risk individuals [22], reduced blood pressure values [23], lower total cholesterol and triglyceride concentrations [3], improved antithrombotic mechanisms [24], and a decreased heart failure risk in cohort studies [25]. MedDiet is known for its richness in antioxidant bioactive compounds, which is thought to be responsible for the decrease in oxidative stress and low-grade inflammation [26,27]. Its high content in monounsaturated and polyunsaturated fatty acids may also be involved in the anti-inflammatory and lipid-lowering effect [28], and its great content in fiber may also be involved in the improvement of several mechanisms involving gut microbiota [28].

We also observed a non-linear association between LTPA and a decreased necessity of cardiovascular drugs. With the exception of vitamin K epoxide reductase inhibitors, we observed the lowest risk of starting to use glucose-lowering, statin, fibrate, antiplatelet, antianginal, and cardiac glycoside medications around 200 METs-min/day of LTPA (the maximum decreases in antihypertensive initiation risk were also associated with values up to 200 METs-min/day). This is equivalent to 45 min of walking at brisk pace or 30 min of jogging [29]. Meta-analyses of human studies have reported that similar values are usually the optimum point in the dose-dependent association between LTPA and the risk of developing a cardiovascular event [2], type 2 diabetes [2], or heart failure [30].

The combination of high LTPA levels and MedDiet adherence was synergistically associated with a delayed initiation of glucose-lowering, antihypertensive, vitamin K epoxide reductase inhibitor, and cardiac glycoside therapy. These findings agree with previous evidence, since the combination of both lifestyle modifications decreases fasting glucose, insulin resistance, blood pressure [12,13], and low-grade inflammation [13] (which could explain the improvement in thrombosis-related responses, related to vitamin K antagonist use). The combination of a healthy hypocaloric diet with exercise has also been additively associated with improvements in heart failure symptoms such as intolerance to physical efforts [31]. However, to the best of our knowledge, this is the first study to report a synergistic association between a healthy, normocaloric diet plus LTPA and improvements in clinical outcomes related to glucose metabolism, blood pressure, thrombosis responses, and heart failure. This synergy could be due to the fact that MedDiet and LTPA enhance these factors through complementary molecular mechanisms. On the one hand, MedDiet has been shown to be able to improve glucose metabolism [32], blood pressure [33], atherothrombosis biomarkers [34], and heart failure indicators [35]. These effects may be partially explained by the capacity of its some of MedDiet bioactive components such as dietary antioxidants to improve oxidative stress and low-grade inflammation [26,27]. Decreases in oxidative stress and low-grade inflammation have been linked to improvements in the molecular sensitivity of insulin in its target cells [36], increased endothelial integrity [37], decreased activation of pro-thrombotic signals [38,39], and better heart dynamics in previous studies [40]. On the other hand, LTPA is known to activate AMP-activated protein kinase through its capacity to produce transient increases in the ratio AMP:ATP [41], which in turn may enhance the previous risk factors by alternative mechanisms. Particularly, it is an enzymatic complex capable of promoting the expression of several metabolic and regulatory proteins such as enzymes involved in glucose metabolism, endothelial nitric oxide synthase, antioxidant enzymes, and downregulators of low-grade inflammation [42,43].

Our study has limitations. First, we conducted our analyses using data of the PREDIMED trial, a dietary intervention study that did not include any LTPA advice. Therefore, we analyzed them as a cohort study, and tried to minimize this limitation by adjusting for the intervention group and by taking as exposure the cumulative averages of MedDiet adherence and LTPA levels. Second, our variables can be considered a proxy for the debut of cardiovascular conditions (a lower initiation of cardiovascular drug use could be due to the fact that there is less need for medication to control the underlying cardiovascular risk factors), but we cannot rule out other less obvious reasons for not using medication (unwillingness/resistance to use pharmacological therapy, etc.). Third, we could only collect categorical information on drug use/non-use and, consequently, we could not study dose changes. Fourth, initiation of cardiovascular-related therapy was not a predetermined endpoint in the PREDIMED study; therefore, our findings should be considered as exploratory and confirmed in further research. Fifth, our conclusions only apply to older individuals at high cardiovascular risk and cannot be generalized to other populations. Finally, some covariates such as alcohol and energy intake are self-reported, which may be prone to misclassification.

## 5. Conclusions

In an older population at high cardiovascular risk, adherence to a MedDiet was associated with lower necessity of cardiovascular-related medications (glucose-lowering, antihypertensive, statin, fibrate, antiplatelet, vitamin K epoxide reductase inhibitor, antianginal, and cardiac glycoside therapies). LTPA levels were also non-linearly related to lower drug initiation, with minimum risks observed around 200 METs-min/day. Finally, the combination of high MedDiet adherence and LTPA levels was synergistically associated with a decreased necessity of glucose-lowering, antihypertensive, vitamin K antagonist, and cardiac glycoside therapies. Our results highlight the potential synergistic association between a healthy diet and LTPA with an improvement in cardiovascular health in individuals at high cardiovascular risk.

## Figures and Tables

**Table 1:** Study population ^1^.

	Main Analytical Sample (*n* = 7063)
Age (years), mean ± SD	67.0 ± 6.2
Female sex, *n* (%)	4080 (57.8)
Diabetes, *n* (%)	3442 (48.7)
Hypercholesterolemia, *n* (%)	5087 (72.0)
Hypertriglyceridemia, *n* (%)	2045 (29.0)
Hypertension, *n* (%)	5834 (82.6)
Smoking habit:	
Never smokers, *n* (%)	4345 (61.5)
Current smokers, *n* (%)	985 (13.9)
Former smokers, *n* (%)	1733 (24.5)
Weight status (according to body mass index):	
18.5–24.9 kg/m^2^, *n* (%)	523 (7.40)
25.0–29.9 kg/m^2^, *n* (%)	3207 (45.4)
≥30 kg/m^2^, *n* (%)	3333 (47.2)
PREDIMED Study intervention groups:	
MedDiet enriched with extra-virgin olive oil, *n* (%)	2465 (34.9)
MedDiet enriched with mixed nuts, *n* (%)	2308 (32.7)
Control group, *n* (%)	2290 (32.4)
MedDiet adherence score, mean ± SD	8.69 ± 1.90
Leisure-time physical activity (metabolic equivalents of task-min/day), median (1st–3rd quartile)	175 (66.1–319)
Alcohol intake (g/day), median (1st–3rd quartile)	1.49 (0.00–10.4)
Energy intake (kcal/day), mean ± SD	2274 ± 604
Carbohydrates (g/day), median (1st–3rd quartile)	227 (182–279)
Protein (g/day), median (1st–3rd quartile)	90.2 (77.0–106)
Total fat (g/day), median (1st–3rd quartile)	96.4 (77.5–116)
Saturated fat (g/day), median (1st–3rd quartile)	24.1 (19.1–30.1)
Monounsaturated fat (g/day), median (1st–3rd quartile)	48.5 (36.4–58.9)
Polyunsaturated fat (g/day), median (1st–3rd quartile)	14.4 (11.0–19.3)
Omega-3 polyunsaturated fat (g/day), median (1st–3rd quartile)	2.04 (1.56–2.68)
Fiber (g/day), median (1st–3rd quartile)	24.0 (19.4–29.9)
Dietary cholesterol (mg/day), median (1st–3rd quartile)	357 (284–429)
Sodium (mg/day), median (1st–3rd quartile)	2263 (1767–2877)
Potassium (mg/day), median (1st–3rd quartile)	4195 (3586–4940)
Calcium (mg/day), median (1st–3rd quartile)	993 (779–1274)
Dietary vitamin D (μg/day), median (1st–3rd quartile)	4.77 (3.33–8.61)
Vitamin C (mg/day), median (1st–3rd quartile)	184 (139–245)
Vitamin E (mg/day), median (1st–3rd quartile)	9.31 (7.57–11.7)

^1^ MedDiet: Mediterranean diet; PREDIMED: PREvención con DIeta MEDiterránea.

## Data Availability

The dataset analyzed during the current study is not publicly available due to national data regulations and for ethical reasons, including that we do not have the explicit written consent of the study participants to make their deidentified data available at the end of the study. However, data described in the manuscript will be made available upon request by sending a letter to the PREDIMED Steering Committee (predimed-steering-committe@googlegroups.com). The request will be passed to all the members of the Committee for deliberation.

## Supplementary Materials

The following are available online at https://www.mdpi.com/2076-3921/10/3/397/s1, Table S1: Power analyses for study determinations, Appendix SI: Full list of PREDIMED study collaborators, Appendix SII: Information and informed consent sheets.
