# Supplementary material for: Mediterranean Diet and Physical Activity Decrease the Initiation of Cardiovascular Drug Use in High Cardiovascular Risk Individuals: A Cohort Study
Source: Antioxidants (Basel). 2021 Mar 5;10(3):397. doi: 10.3390/antiox10030397 (PMC7999777; doi:10.3390/antiox10030397)
Supplement: Supplementary file 1 [file antioxidants-10-00397-s001.zip › supplementary file antioxidants-1108330/Appendix SI.docx]

**APPENDIX SI**

**Full list of PREDIMED study collaborators**

Hospital Clinic, Institut d’Investigacions Biomèdiques August Pi i Sunyer, Barcelona, Spain: R. Estruch, M. Serra, A. Pérez-Heras, C. Viñas, R. Casas, L. de Santamaría, S. Romero, E. Sacanella, G. Chiva, P. Valderas, S. Arranz, J.M. Baena, M. García, M. Oller, J. Amat, I. Duaso, Y. García, C. Iglesias, C. Simón, L. Quinzavos, L. Parra, M. Liroz, J. Benavent, J. Clos, I. Pla, M. Amorós, M.T. Bonet, M.T. Martin, M.S. Sánchez, J. Altirriba, E. Manzano, A. Altés, M. Cofán, C. Valls-Pedret, A. Sala-Vila, M. Doménech, R. Gilabert, and N. Bargalló.

University of Navarra, Primary Care Centres, Pamplona, Spain: M.Á. Martínez-González, A. Sánchez-Tainta, B. Sanjulián, E. Toledo, M. Bes-Rastrollo, A. Martí, C. Razquin, P. Buil-Cosiales, M. Serrano-Martínez, J. Díez-Espino, A. García-Arellano, I. Zazpe, F.J. Basterra-Gortari, E.H. Martínez-Lapiscina, A. Gea, M. Garcia-Lopez, J.M. Nuñez-Córdoba, N. Ortuño, N. Berrade, V. Extremera-Urabayen, C. Arroyo-Azpa, L García-Pérez, J. Villanueva-Tellería, F. Cortés-Ugalde, T. Sagredo-Arce, Mª D. García de la Noceda-Montoy, Mª D. Vigata-López, Mª T. Arceiz-Campo, A. Urtasun-Samper, Mª V. Gueto-Rubio, and B. Churio-Beraza.

University of Valencia, Valencia, Spain; Universitat Jaume I, Castellon, Spain; and Conselleria de Sanitat, Generalitat Valenciana: D. Corella, P. Carrasco, C. Ortega-Azorín, E.M. Asensio, R. Osma, R. Barragán, F. Francés, M. Guillén, J.I. González, C. Sáiz, O. Portolés, F.J. Giménez, O. Coltell (U. Jaume I), R. Fernández-Carrión, I. González-Monje, L. Quiles, V. Pascual, C. Riera, M.A. Pages, D. Godoy, A. Carratalá-Calvo, S. Sánchez-Navarro, and C. Valero-Barceló.

University Rovira i Virgili, Reus, Spain: J. Salas-Salvadó, M. Bulló, R. González, C. Molina, F. Márquez, N. Babio, M. Sorlí, J. García-Roselló, F. Martin, R. Tort, A. Isach, B. Costa, J.J. Cabré, J. Fernández-Ballart, N. Ibarrola, C. Alegret, P. Martínez, S. Millán, J.L. Piñol, J. Basora, and J.M. Hernández.

Institut Hospital del Mar d’Investigacions Mèdiques, Barcelona, Spain: M. Fitó, M.I. Covas, O. Castañer, S. Tello, J. Vila, H. Schröder, R. De la Torre, D. Muñoz-Aguayo, N. Molina, E. Maestre, A. Rovira, R. Elosua, and M. Farré.

University Hospital of Alava, Vitoria, Spain: F. Arós, I. Salaverría, T. del Hierro, J. Algorta, S. Francisco, A. Alonso-Gómez, J. San-Vicente, E. Sanz, I. Felipe, A. Alonso-Gómez, and A. Loma-Osorio.

University of Málaga, Málaga, Spain: E. Gómez-Gracia, R. Benítez-Pont, M. Bianchi-Alba, J. Fernández-Crehuet Navajas, J. Wärnberg, R. Gómez-Huelgas, J. Martínez-González, V. Velasco-García, J. de Diego-Salas, A. Baca-Osorio, J. Gil-Zarzosa, J.J. Sánchez-Luque, and E. Vargas-López.

Institute of Health Sciences, University of Balearic Islands, and Hospital Son Espases, Palma de Mallorca, Spain: M. Fiol, M. García-Valdueza, M. Moñino, A. Proenza, R. Prieto, G. Frontera, M. Ginard, F. Fiol, A. Jover, and J. García.

Department of Family Medicine, Primary Care Division of Sevilla, Sevilla, Spain: J. Lapetra, M. Leal, E. Martínez, J.M. Santos, M. Ortega-Calvo, P. Román, F.J. García, P. Iglesias, Y. Corchado, E. Mayoral, and C. Lama.

University of Las Palmas de Gran Canaria, Las Palmas, Spain: L. Serra-Majem, J. Álvarez-Pérez, E. Díez-Benítez, I. Bautista-Castaño, I. Maldonado-Díaz, A. Sánchez-Villegas, F. Sarmiendo-de la Fe, C. Simón-García, I. Falcón-Sanabria, B. Macías-Gutiérrez, and A.J. Santana-Santana.

Hospital Universitario de Bellvitge, Hospitalet de Llobregat, Barcelona, Spain: X. Pintó, E. de la Cruz, A. Galera, Y. Soler, F. Trias, I. Sarasa, E. Padres, R. Figueras, X. Solanich, R. Pujol and E. Corbella.

Clinical End Point Committee: F. Arós (chair), M. Aldamiz, A. Alonso-Gómez, J. Berjón, L. Forga, J. Gállego, M. A. García-Layana, A. Larrauri, J. Portu, J. Timiraus, and M. Serrano-Martínez.
