# Supplementary material for: Mediterranean Diet and Physical Activity Decrease the Initiation of Cardiovascular Drug Use in High Cardiovascular Risk Individuals: A Cohort Study
Source: Antioxidants (Basel). 2021 Mar 5;10(3):397. doi: 10.3390/antiox10030397 (PMC7999777; doi:10.3390/antiox10030397)
Supplement: Supplementary file 1 [file antioxidants-10-00397-s001.zip › supplementary file antioxidants-1108330/Appendix SII. Information and informed consent sheets.pdf]

**APPENDIX SII. Information and informed consent sheets**

## PATIENT INFORMATION SHEET

**Project title:** EFFECTS OF THE MEDITERRANEAN TYPE DIET ON THE PRIMARY PREVENTION OF CARDIOVASCULAR DISEASE

**Participant(s) and Principal Investigator(s):**
- (Write down name)

### Objective

To investigate the effect of a Mediterranean-type diet supplemented with virgin olive oil or nuts and dried fruit on the prevention of cardiovascular disease in patients at high risk of suffering from it, comparing it with the usually recommended diet.

### Methodology used. Development of the study

A total of 16 centers throughout Spain are participating in this study and the intention is to include a total of 12,000 patients who present various vascular risk factors.
Once included in the study:

- A medical history will be taken and surveys will be conducted on your food consumption, physical activity and consumption of tobacco and alcohol.
- A physical examination will be performed with measurements of weight, height, and waist and hip diameter.
- Blood will be drawn (about 40 mL) and a urine sample and a small fragment of your toenails will be collected.
- An electrocardiogram will be performed.
- You will be advised about the diet you should follow and every 3 to 6 months, you will be insisted on that diet. In addition, some participants will receive some supplements of nuts or virgin olive oil.

These tests will be repeated annually until the study is completed.
The expected duration of the study is 3 years, although it is possible to extend it to 5 years. It is also possible that the Monitoring Committee will decide the early termination of the study based on provisional results.

### Intervention

This project aims to evaluate the effect of a Mediterranean type diet against the usually recommended diet. One third of the participants will receive the usual dietary advice and the remaining two thirds will be advised on a Mediterranean-type diet (rich in vegetables, fruits, vegetables, fish, poultry, legumes, etc.). In addition, some of the participants included in the Mediterranean-type diet group will receive supplementary amounts of nuts (walnuts, almonds, hazelnuts) and others will receive supplements of virgin olive oil. Entering into one or the other group will be by chance. You will not be given any specific medication, nor will your usual treatment be modified. If throughout the study modifications in your usual treatment were necessary, these will be carried out by the doctor/s who usually attend you.

### Benefits and risks

Your participation in the study can help you to better understand your health status, cardiovascular risk factors and a healthy diet. It is also possible that you will not gain any direct benefit from participating in the study. However, it is likely that some of the information obtained may benefit other patients in the future and may contribute to a better understanding of the effect of diet on cardiovascular disease.
The study does not involve any risk to your health as the amount of blood drawn will be slightly higher than the amount drawn when performing a complete blood test. The extraction of the blood sample may cause a burning sensation at the point where the needle is inserted into the skin and may cause a small bruise that disappears in a few days. More rarely, it can cause temporary dizziness.

**Adverse events**

As this is not a study with drugs, no adverse effects are expected, except for some hypersensitivity reaction to some of the components of the Mediterranean diet such as olive oil or nuts, which you have probably already tried.

**Volunteers**

Your participation in this study is completely voluntary so you can withdraw from it at any time, without having to give any explanation and without affecting your relationship with your usual medical team.

**Confidentiality**

As provided in Regulation (EU) 2016/679 of the European Parliament and Council of April 27, 2016 on the protection of individuals with regard to the processing of personal data and on the free movement of such data and repealing Directive 95/46/EC (General Data Protection Regulation), all data collected about your participation in this study will be considered confidential. The data collected for the study will be identified by a code, so that no information is included that allows your identification, and only your study doctor/collaborators will be able to relate this data to you and your medical history. Therefore, your identity will not be disclosed to anyone except health authorities, when required, or in cases of medical emergency. Research Ethics Committees, health authority inspection representatives, and personnel authorized by the Sponsor may only have access to these data for the purpose of checking personal data, clinical trial procedures, and compliance with good clinical practice standards, while maintaining confidentiality of the information. The results will be analyzed by groups or subgroups of investigators. The absolute confidentiality of the data obtained in the study is guaranteed. The work lists will not include your name and will only include the number assigned to you in the study. In the final report of the study or in case of communicating these results to the scientific community, your personality will remain anonymous. The data will be kept for a period of 15 years after the publication of the results derived from the research, as specified in the applicable regulations (Law 14/2007 on Biomedical Research (LIB) in studies with biological samples).
These data may be subject to automated processing and the rights of study participants to consult, modify or delete their personal data from the file. The responsibility for the global file corresponds to the Hospital Clínic de Barcelona, but the different participating centers will also have a copy of the global data of the study.
In addition to the rights that you already know (access, modification, opposition and cancellation of data), you can now also limit the processing of data that is incorrect, the

request for a copy or the transfer to third parties (portability) of those information that you have provided throughout the study. To exercise your rights, you should contact the principal investigator of the study or the Data Protection Delegate of the center (protecciodedades@imim.es). We remind you that the data cannot be deleted even when you leave the study in order to guarantee the validity of the research and to comply with legal responsibilities. You also have the right to file complaints with the relevant data authority in Spain, the Data Protection Agency, whose contact details can be found at www.agpd.es.

**Compensation**
No financial compensation is envisaged. Volunteers assigned to the intervention group with a Mediterranean diet enriched with virgin olive oil or nuts will receive these nutritional products to encourage compliance with the interventions, while those assigned to the control group will receive an equivalent non-pecuniary reward, so that all volunteers receive the same treatment throughout the study.

**Study investigators**
If you have any questions about any aspect of the study or would like to comment on any aspect of this information, please do not hesitate to let the members of the research team at your hospital (Drs ................................, ................................ or the Coordinating Center, Dr R. Estruch, Tel 93 2279365. In case that once you have read this information and clarified your doubts you decide to participate in the study, you must sign your informed consent.
This study was approved by the Clinical Research Ethics Committee of Hospital del Mar de Barcelona.

**INFORMED CONSENT FORM**

**Project title:** EFFECTS OF THE MEDITERRANEAN TYPE DIET ON THE PRIMARY PREVENTION OF CARDIOVASCULAR DISEASE

**Participant(s) and Principal Investigator(s):**
- (Write down name)

I,..........................................................................................
(name and surname)

- I have read the information sheet I was given.
- I have been able to ask questions about the study
- I have received enough information about the study

I have spoken to ..........................................................................
(name of researcher)

I understand that my participation is voluntary.
I understand that I may withdraw from the study:
- at any time.
- without having to give explanations
- without affecting my medical care

I agree to participate in the study

| | |
|---|---|
| ______________________ | ______________________ |
| Date | Participant's signature |
| ______________________ | ______________________ |
| Date | Researcher's signature |
