# Supplementary material for: Mediterranean Diet and Physical Activity Decrease the Initiation of Cardiovascular Drug Use in High Cardiovascular Risk Individuals: A Cohort Study
Source: Antioxidants (Basel). 2021 Mar 5;10(3):397. doi: 10.3390/antiox10030397 (PMC7999777; doi:10.3390/antiox10030397)
Supplement: Supplementary file 1 [file antioxidants-10-00397-s001.zip › supplementary file antioxidants-1108330/Table S1.docx]

**Supplemental Table S1.** Power analyses for study determinations

|  | **Low adherence to a MedDiet + low physical activity levels** | | **High adherence to a MedDiet + high physical activity levels** | | **HR detectable**  **with ≥80% power** | |
| --- | --- | --- | --- | --- | --- | --- |
|  | Number  of cases | Total  individuals | Number  of cases | Total  individuals | <1 | >1 |
| Glucose-lowering drug use | 251 | 1,280 | 164 | 1,272 | ≤0.76 | ≥1.32 |
| Antihypertensive drug use | 340 | 626 | 210 | 601 | ≤0.78 | ≥1.27 |
| Statin use | 391 | 1,156 | 283 | 1,105 | ≤0.80 | ≥1.24 |
| Fibrate use | 51 | 1,868 | 29 | 1,834 | ≤0.53 | ≥1.87 |
| Antiplatelet drug use | 350 | 1,667 | 252 | 1,660 | ≤0.79 | ≥1.26 |
| Vitamin K antagonist use | 74 | 2,005 | 48 | 1,941 | ≤0.60 | ≥1.66 |
| Antianginal drug use | 62 | 1,992 | 42 | 1,933 | ≤0.57 | ≥1.73 |
| Cardiac glycoside use | 23 | 2,020 | 11 | 1,971 | ≤0.38 | ≥2.60 |

*HR:* hazard ratio; *MedDiet*: Mediterranean diet.
